# 

**Published:** 1910-02-26

**Authors:** 


### MANCHESTER ROYAL INFIRMARY.

**The Institution Library.**

"Hospital Expenditure: The Commissariat." For the use of Managers. ... ... ... ... ... 2s. 6d.
"The District Nursing Association; Hints on how to Start it." (Net, post free.) ... ... ... 1s 1d.
"A Legal Handbook for the use of Hospital Authorities." ... ... ... ... ... ... ... 2s. 6d.
Ledgers, Charts, Case-papers, and every kind of Institutional Literature.

Of all booksellers or of The Scientific Press, Limited, 28 & 29 Southampton Street, Strand, London, W.C.

### Gifts and Bequests.

The Treasurer of Guy's Hospital has received £1,000 from Mr. R. Nivison.

Mr. Edward Bell, of Wray Park, Reigate, has left £100 to the Sussex County Hospital.

Mr. William Hilton, of Oldham, Lancashire, has left £1,000 to the Oldham Infirmary.

Mr. James Hunter, of Peterculter, Aberdeen, has left, free of estate duty, £100 to the Aberdeen Royal Infirmary.

The Queen's Hospital for Children, Hackney Road, has received £52 10s. from the Mercers' Company and £50 from the Grocers' Company.

The Committee for the removal of King's College Hospital to South London has received £500 from the trustees of the late Lord Grimthorpe's Charity Fund, and also a second donation of 100 guineas from the trustees of the late Robert William Edwards.

Miss Annette Harding, of Leamington, Warwick, has left £100 each to the Birmingham General Hospital and the Birmingham Children's Hospital; £50 to the Warneford Hospital, Leamington; and £25 to the Provident Dispensary, 20 Lower Holly Walk, Leamington.

Special donations of £500 each were announced at the centenary meeting of the Taunton and Somerset Hospital from Lord Portman, the Hon. E. W. B. Portman (who was re-elected President), Mrs. Alexander Hammett, of Taunton, in memory of her late husband, and Mrs. Lucas, of Tolland Rectory, out of the residuary estate of her uncle, the late Mr. William Bashall Park, of Ollerton Hall, near Chorley, Lancashire.

Mrs. Jane Hannah Thomson, of Culverden Park, Tunbridge Wells, has left £1,000 to the Royal Hospital for Incurables, Putney, £500 to the Tunbridge Wells General Hospital, and the residue of her estate, subject to other legacies and the payment of duties, to the Royal Hospital for Incurables, Putney, and two other institutions. The amount available for charitable purposes under Mrs. Thomson's will appears to be about £30,000.

### THE MEDICAL LIBRARY.

Post Free.

"Medical History: From the Earliest Times." By E. T. Withington, M.A., M.B. Oxon ... 12s. 6d.

"The Consumptive Working Man. What Can the Sanatoria Do for Him?" By Noel D. Bardswell, M.D. ... ... ... ... ... ... 10s. 10d.

"Photomicrography." By Edmund Spitta, M.R.C.S. With 41 Half-tone Reproductions from original negatives and other Illustrations ... ... 12s. 0d.

"Outlines of Insanity." A Popular Treatise on the Salient Features of Insanity. ... ... ... 1s. 6d.

"Sight and Hearing in Childhood." By R. Brudenell Carter, F.R.C.S., and Arthur Cheatle, F.R.C.S. 2s. 3d.

Of all booksellers or of The Scientific Press, Limited, 28 & 29 Southampton Street, Strand, London, W.C.

### Appointments.

Mr. Archibald Kidd, M.R.C.S., D.P.H., who has been acting as temporary assistant medical officer to the Port of London, has now been permanently appointed to that office.

Mr. Percy Flemming, M.D., B.S., F.R.C.S., has been appointed Lecturer in Ophthalmology at the London School of Medicine for Women, in succession to the late Miss Ellaby, M.D.

The Board of Management of the Manchester Royal Infirmary has made the following appointments :—Senior house surgeons : T. W. Todd, M.B., Ch.B. (Vict.); E. E. Hughes, M.B., Ch.B. (Vict.). Junior house surgeons : N. T. K. Jordan, M.R.C.S., L.R.C.P. ; C. T. H. Newton, M.B., Ch.B. (Edin.). All appointed for a period of six months from March 1 next.

### THE QUESTION BOX.

**Special Notice.**—Questions of interest affecting the honorary medical staff and hospital administration in all departments are invited. When any point arises we hope our readers will formulate it in a question and so co-operate to make this column of real value and interest. In this way the experience of the best-managed hospitals should be made helpful to the whole hospital world. We shall welcome the contribution of both questions and answers.

**N.B.**—The publication of an answer in this column does not necessarily preclude the publication of subsequent answers to the same question, for a question may involve points which require to be threshed out and fully discussed. Answers, if published, will be paid for at scale rates.

### A CENTRAL STORE FOR HOSPITALS.

### Not Feasible for Greater London Hospitals.

By Mr. C. W. Thies, Secretary Royal Free Hospital.

The question of establishing some kind of central store for supplying, on co-operative principles, the various articles required by our voluntary hospitals has from time to time engaged the attention of many persons who are responsible for the administration of these institutions. The advantage to be derived from any such arrangement is almost entirely confined to the economy in purchasing supplies, which it is supposed must result from buying the various articles in large quantities. In theory this appears to be a simple and self-evident proposition, but, when all the circumstances of the case are taken into consideration, it will be seen that the problem, as applied to the hospitals of London, is not by any means as simple as at first sight it appears to be. In such cities as Paris, where the hospitals generally are administered by a central authority, it is a comparatively easy matter to organise a central store, from which all supplies are issued as required by the various hospitals. The Assistance Publiqué of Paris, indeed, undertakes this work, providing its constituent institutions with nearly all their requirements, including such perishable articles as milk, bread, etc.

In endeavouring to apply this method of obtaining the necessary supplies to our London voluntary hospitals we must take into consideration the actual conditions, which for brevity's sake I venture to summarise as follows :—

1. The voluntary hospitals of London are constant and active competitors for financial support, and their interests are not by any means identical.

2. There is great diversity in their special requirements owing to the different character of their work, size, and methods.

3. The establishment of a central store, with adequate accommodation and equipment, the necessary and costly large stocks which must be kept in hand, must involve a very heavy capital outlay. Where is this capital to come from?

4. The cost of maintenance of such a store, including rent, distributing, and other up-keep charges, together with the salaries of expert buyers, would be considerable and must add very materially to the prime cost of the various articles. Would the saving in prices be sufficient to cover these expenses?

5. It can hardly be expected that the manufacturers, merchants, and middle men, who at present compete with each other for the orders of the hospitals, very often at a very low margin of profit, would readily acquiesce in any such scheme; for instance, take the important item of coal.

I am of opinion that under existing conditions the only method of co-operation at present practicable is for some of the smaller hospitals to combine for the purpose of obtaining better terms for the supply of certain staple articles.

There are several other aspects of the proposal which could be mentioned, but I leave them to be dealt with by your other comtributors.

### SUCCESSFUL ACTION FOR FEES.

In the Lord Mayor's Court this week a claim was made by Mr. Vincent Warren Low, consulting surgeon, against Mr. Adolphus M. Seymour, a solicitor, of Staines, for £34 for fees in respect of an operation performed last year upon the defendant's wife. The plaintiff's case was that in April last he was rung up on the telephone by Dr. Batchelor, practising at Staines, asking him to see a lady patient suffering from an internal malady. He agreed to attend, and upon examination of the patient found she was in a serious condition, and but for an immediate operation the patient could not live more than a few hours. An operation was performed for which a fee of fifty guineas was charged, and then it was discovered that there was a growth. After the operation a question arose as to the necessity of a further operation for the removal of the growth. This it was eventually arranged should be carried out, the fee charged to be thirty guineas. The second operation was performed, the defendant being informed that it was a risky one, but that the patient would probably pull through, unless complications ensued. The patient rallied for a few weeks and then peritonitis ensued, from which she died. Mr. Low had consented to wait for the payment of his second fee upon the defendant stating that the matter was an expensive business to him, but after waiting six months applied for the balance of his fee for the first operation, the defendant having paid £50 instead of the agreed fee of fifty guineas, and also the thirty guineas. Mr. Seymour then put forward more definitely certain charges of negligence against the plaintiff. Plaintiff denied there was such negligence or that there was anything that could be done surgically that was not done. He had not admitted to Dr. Batchelor that there had been an omission by him in carrying out the first operation which would be a lesson to him for the rest of his life, and he had not mentioned to the defendant the sum of £50 as a settlement of account for all the operations.

After hearing the evidence the jury returned a verdict for the plaintiff on the claim for £34, and some of the jurors said they wished to express their sympathy with the defendant.

The Duke of Buccleuch, who recently gave a public park to Dalkeith, has, it is announced, offered some acres of ground at Whitehill for a hospital for infectious diseases. He has also intimated that he will give a donation towards its erection, as the present hospital at Newmills is unsatisfactory, and does not admit of extensions which the Local Government Board is urgently calling for.

The Orient Company announces a pleasure cruise to Portugal, Spain, Morocco, Gibraltar, Algeria, the Balearic Islands, and Marseilles by the s.s. *Ormuz* from London to Greece, Asia Minor, Turkey, Egypt, and Malta on March 12. On May 13 the new twin-screw s.s. *Otranto*, 12,124 tons register, will cruise to Portugal, Balearic Islands, Spain, Morocco, and Gibraltar. This vessel's accommodation is of the most palatial character, with cabines de luxe, spacious lounges, and electric elevators. An illustrated programme will be sent on application to the company, 5 Fenchurch Avenue, London, E.C.

THE

### GRADUATES' MEDICAL SCHOOLS.

DIARY FOR THE WEEK, FEB. 28 to MARCH 5.

**MEDICAL SOCIETY OF LONDON, Chandos Street, Cavendish Square, W.**

**At 8.30 p.m.—Feb. 28,** Sir J. Broadbent, Bart., M.D., **Some Difficulties in Prognosis in Cases of Atypical Pyrexia in Pneumonia in Adults.**

**8.30 p.m.—Feb. 28,** Dr. Reginald Miller, **Some Cases of Protracted Pyrexia in Pneumonia in Children.**

**LONDON SCHOOL OF CLINICAL MEDICINE, Seamen's Hospital, Greenwich, S.E.**

**At 4 p.m.—Feb. 28,** Mr. R. Lake, **Operations in Tubercle of the Larynx.**

**3.15 p.m.—March 1,** Mr. Carless, **Actinomycosis.**

**3.30 p.m.—March 2,** Mr. Cargill, **Ophthalmoscopic Appearances in Typical Diseases of the Fundus Oculi.**

**NORTH-EAST LONDON POST-GRADUATE COLLEGE, Prince of Wales's General Hospital, Tottenham, N.**

**At 4.30 p.m.—March 1,** Dr. A. J. Whiting, **Colitis and Pericolitis.**

**CENTRAL LONDON THROAT AND EAR HOSPITAL, Gray's Inn Road, W.C.**

**At 3.45 p.m.—March 1,** Mr. Stuart Low, **Accessory Sinuses.**

**4.30 p.m.—March 3,** Mr. Gay French, **Suppuration of the Accessory Sinuses.**

**3.45 p.m.—March 4,** Mr. Stuart Low, **Accessory Sinuses**

**THE BROMPTON HOSPITAL FOR CONSUMPTION AND DISEASES OF THE CHEST, Fulham Road, S.W.**

**At 4 p.m.—March 2,** Dr. Batty Shaw, **Open and Closed Pulmonary Tuberculosis.**

**ST. JOHN'S HOSPITAL FOR DISEASES OF THE SKIN, Leicester Square, W.C.**

**At 6 p.m.—March 3,** Chesterfield Lecture: **Fungous Diseases of the Hair.**

**MEDICAL GRADUATES' COLLEGE AND POLYCLINIC, 22 Chenies Street, W.C.**

**At 4 p.m.—Feb. 28,** Dr. Graham Little, **Clinique (skin).**

**5.15 p.m.—Feb. 28,** Mr. A. W. Mayo Robson, **The Surgical Treatment of Certain Cases of Glycosuria.**

**4 p.m.—March 1,** Dr. Leonard Guthrie, **Clinique (med.).**

**5.15 p.m.—March 1,** Mr. E. Laming Evans, **The Manipulative Treatment of Congenital Dislocation of the Hip.**

**4 p.m.—March 2,** Mr. A. Pearce Gould, **Clinique (surg.)**

**5.15 p.m.—March 2,** Dr. E. F. Bashford, **The Results of Experimental Cancer Research.**

**4 p.m.—March 3,** Sir Jonathan Hutchinson, **Clinique (surg.).**

**5.15 p.m.—March 3,** Mr. Clinton Dent, **After Effects of Head Injuries.**

**4 p.m.—March 4,** Mr. Hunter Tod, **Clinique (ear, nose, and throat).**

**THE POST-GRADUATE COLLEGE, West London Hospital, Hammersmith, W.**

**At 10 a.m.—Feb. 28, Demonstration by Surgical Registrar.**

**10 a.m.—March 4, Medical Registrar, Demonstration of Cases.**

**5 p.m.—Feb. 28,** Mr. Lloyd Williams, **Examination and Treatment of Children's Teeth.**

**5 p.m.—March 1,** Dr. Seymour Taylor, **Cerebral Hæmorrhage.**

**5 p.m.—March 2,** Dr. R. Morton, **X-Ray Diagnosis.**

**5 p.m.—March 3,** Mr. Baldwin, **Practical Surgery.**

**5 p.m.—March 4,** Mr. Etherington-Smith, **Clinical Lecture.**

**ROYAL SOCIETY OF MEDICINE, 20 Hanover Square, W.**

**At 8 p.m.—Feb. 28, Odontological Section:**

*Paper.*—Mr. Stanley P. Mummery: "Some experiments on the relative susceptibility of different teeth to dental caries."

*Casual Communications.*—Dr. Charles A. Clark: (1) "A sterilisable film-holder for *x*-ray work"; (2) "A method of ascertaining the relative position of unerupted teeth by means of film radiographs."

**At 4.30 p.m.—March 1, Therapeutical and Pharmacological Section:**

*Adjourned Discussion* on "Proprietary, patent, and secret medicines," opened by Prof. W. E. Dixon, on February 1.

*Paper.*—Dr. John Cowan: "The treatment of infective endocarditis."

**At 5 p.m.—March 4, Laryngological Section:**

*Cases and Specimens* will be shown by the following: Dr. A. Wylie, Dr. S. Moritz, Mr. Herbert Tilley, Dr. A. R. Tweedie, Mr. Somerville Hastings, Dr. E. A. Peters, Mr. Harold Barwell, Mr. Chichele Nourse, and others.

**At 8.30 p.m.—March 4, Section of Anæsthetics:**

*Discussion* on "The choice of an anæsthetic," opened by Dr. Dudley Buxton.

**At 10 a.m.—March 5, Otological Section:**

*Cases and Specimens.*—Mr. A. H. Cheatle: Twenty specimens of chronic middle-ear suppuration and its sequelæ. Mr. W. Milligan: (1) Pedunculated papilliform growth springing from the posterior border of the cartilaginous meatus; (2) Large naso-pharyngeal growth in a boy; (3) Demonstration of Kuhn's instruments for per-oral intubation. Dr. Dan McKenzie: (1) Case with well-defined and transitory Menière's symptoms; (2) ? Othæmatoma. Dr. Alexander Sharp: Audible tinnitus. Mr. J. Arnold Jones: Mucous polypus presenting at the pharyngeal orifice of the left Eustachian tube in a man suffering from bilateral chronic adhesive otitis media. Mr. Hunter Tod: (1) Osteo-myelitis of temporal bone; (2) Subdural abscess and excephalitis of the left temporo-sphenoidal lobe, with aphasia.

